# Humans recognize affective cues in primate vocalizations: acoustic and phylogenetic perspectives

**DOI:** 10.1038/s41598-023-37558-3

**Published:** 2023-07-05

**Authors:** C. Debracque, K. E. Slocombe, Z. Clay, D. Grandjean, T. Gruber

**Affiliations:** 1 Department of Psychology and Educational Sciences and Swiss Center for Affective Sciences (CISA), Campus Biotech, University of Geneva, Chemin des Mines 9, 1202 Geneva, Switzerland; 2 Department of Psychology, University of York, York, UK; 3 Department of Psychology, Durham University, Durham, UK

**Keywords:** Evolution, Human behaviour

## Abstract

Humans are adept at extracting affective information from vocalizations of humans and other animals. However, the extent to which human recognition of vocal affective cues of other species is due to cross-taxa similarities in acoustic parameters or the phylogenetic closeness between species is currently unclear. To address this, we first analyzed acoustic variation in 96 affective vocalizations, taken from agonistic and affiliative contexts, of humans and three other primates—rhesus macaques (*Macaca mulatta*), chimpanzees and bonobos (*Pan troglodytes* and *Pan paniscus*). Acoustic analyses revealed that agonistic chimpanzee and bonobo vocalizations were similarly distant from agonistic human voices, but chimpanzee affiliative vocalizations were significantly closer to human affiliative vocalizations, than those of bonobos, indicating a potential derived vocal evolution in the bonobo lineage. Second, we asked 68 human participants to categorize and also discriminate vocalizations based on their presumed affective content. Results showed that participants reliably categorized human and chimpanzee vocalizations according to affective content, but not bonobo threat vocalizations nor any macaque vocalizations. Participants discriminated all species calls above chance level except for threat calls by bonobos and macaques. Our results highlight the importance of both phylogenetic and acoustic parameter level explanations in cross-species affective perception, drawing a more complex picture to the origin of vocal emotions.

## Introduction

Vocal communication of affect is crucial for the emotional and attentional regulation of human social interactions^1–4^. For instance, the modulation of prosodic features in human speech, such as intonation or amplitude can convey subtle affective information to receivers^5, 6^. Humans consistently recognize and evaluate the affective cues of others’ vocal signals in tasks with varying levels of complexity; with emotion categorization (i.e. unbiased choice, such as A versus B) being apparently more cognitively complex than discrimination (i.e. biased choice, such as A versus non-A)^7, 8^. In both emotion categorization and discrimination tasks, research shows that listeners can subjectively attribute the speaker’s reported affective state (i.e. angry, fearful or happy) as well as any potentially referential content^5, 9^. By no means uniquely human, these affective identification mechanisms can facilitate adaptive behavior in animals, such as avoidance of or approach towards a stimulus^10–13^. It is possible that mechanisms underlying perception of vocalizations are similar in humans and other animals and shaped by similar adaptive pressures. For instance, research has shown in both humans and great apes, receivers use the acoustic roughness of fear screams to rapidly appraise danger^14, 15^. Nevertheless, despite the adaptive value and importance of auditory affective processing to our own species, its evolutionary origins remain poorly understood.

As noted over a century ago by Darwin^16^, there appear to be strong evolutionary continuities between humans and other animals for the vocal expression of affective signals. In his seminal work, Morton^17, 18^ proposed a model of motivational structural rules to characterize the relationship between the acoustic structure of mammal and bird vocalizations and their presumed affective contents. The systematic modulation of call acoustic structure and the caller’s underlying affective state appear to provide reliable cues that allow listeners to evaluate aspects of the eliciting stimulus, such as the level of threat or danger^19, 20^. Comparative research has since confirmed that conspecifics are sensitive to such cues, with playback studies showing that both chimpanzees and rhesus macaques discriminate between agonistic screams produced by victims facing varying degrees of threat^21, 22^. Similarly, meerkats extrapolate the degree of urgency required from the acoustic structure of conspecific alarm calls^23^. This evidence suggests an evolutionary continuity in the vocal processing ability of both humans and non-human primates to accurately identify affective cues in conspecific vocalizations^24^.

Interestingly, this evolutionary continuity is further suggested by a second line of research, which shows highlights human participants apparent ability to identify primate signals. Despite a limited number of published studies (eight to our knowledge)^20, 25–32^, existing findings on human perception of arousal and valence in non-human primate calls are promising. Indeed, research has shown that humans can accurately discriminate the valence of chimpanzee vocalizations, including agonistic screams (negative valence) and food-associated calls (positive valence)^27, 28^; by comparison however, behavioral discrimination for rhesus macaque calls given in the same contexts is poor^25, 27^. Functional Magnetic Resonance Imaging (fMRI) measures, taken by Fritz and collaborators, also showed that neural activations were more similar when attending to chimpanzee and human vocalizations as compared to macaque calls. In contrast, Linnankoski and colleagues^30^ found that both human adults and infants could categorize affective macaque vocalizations in a larger range of contexts (angry, fearful, satisfied, scolding and submissive). Methodological differences might explain these differences: it may be easier to label affective contents of non-human primate vocalizations in a forced choice paradigm (categorization or discrimination tasks) in which the number of possibilities is limited rather than to rate the valence or arousal using Likert scales. For instance, research with human affective stimuli using forced choice paradigms has demonstrated the positive relationship between cognitive complexity and the number of available categories to choose from^7, 8^. Thus, forced choice paradigms with limited options to choose from may lead to elevated performance in identifying macaque calls^30^ compared to Likert rating scales^25, 27^.

In addition to the mixed findings concerning human sensitivity to valence in non-human primate vocalizations, evidence that humans can accurately judge vocal arousal in other species is also mixed. Recent findings highlight the ability of humans to reliably identify arousal in barbary macaque vocalizations expressed in negative contexts^20^; moreover, arousal ratings of chimpanzee vocalizations seem to be fairly accurate across both positive and negative valences^28^. Nevertheless, Kelly and collaborators^29^ also showed that human participants over-estimated the distress content of bonobo infant calls compared to those of human or chimpanzee ones, suggesting identification of bonobo vocal arousal was more challenging. Overall, humans appear to perform relatively well with chimpanzee calls^28^, but less well with bonobo or macaque calls. Though this requires further investigation, this may be in part be explained by the elevated pitch of bonobo vocalizations, which can be up to one octave higher than those of chimpanzees^33^.

More broadly, several factors might explain variation in the human ability to recognize other species’ affective vocalizations. In line with some evidence from comparative research^34^, previous studies comparing human responses to closely and distantly related species, have highlighted the importance of phylogenetic proximity in human recognition of affect^25, 27^, with humans being more sensitive to emotional content of vocalizations in closely related species. An important test of this hypothesis is to examine responses to vocalizations of two ape species that are equally closely related to us, chimpanzee and bonobos^35^. Although there appears to be a difference in ratings of distress intensity in bonobo infant calls as compared to chimpanzee infant calls^29^, whether this pattern generalizes beyond distress calls is currently unknown.

In addition to phylogenetic proximity, another important factor determining human accuracy at detecting the emotional content of other species’ vocalizations may be their acoustic distance to those of humans, i.e. their closeness in term of acoustic structure reflecting mechanisms of vocal production. More specifically, previous research has linked the human ability to recognize vocal affective cues of other species to specific modulations of the fundamental frequency (F0), the mean pitch, the spectral center of gravity or the energy of their affective calls^20, 30, 31, 36, 37^. In respect to such features, comparative research has highlight similarities in the acoustic communication of affect of closely related species^31, 38^. Nevertheless, despite being closely related to one another and as equally related to us^39^, the vocal repertoire of bonobos shows some notable acoustic differences, including elevated pitch^40^ potentially due to shorter vocal tracts^33^. Hence, it seems reasonable to hypothesize that acoustic differences in bonobo calls may lead to lower performance in a human recognition task.

A key question is the extent to which these two accounts, phylogenetic distance and acoustic distance, can be differentiated, considering that closely phylogenetically-related species are likely to also share vocalizations that are acoustic similar. To the extreme, as closely related species are likely to share similarities in their vocal repertoires, a foreseeable consequence is that humans should recognize more of the affective content of vocalizations if such states are acoustically expressed similarly in humans. In this respect, participants may judge chimpanzee calls as those that sound most like human calls. Given suggested differences in their acoustic pitch, yet comparable phylogenetic relatedness, the addition of bonobos as a comparison group adds a crucial aspect to disentangle the respective contributions of each factor. This is important because if it is shown that participants similarly recognize chimpanzee and bonobo calls, despite their acoustic distance, and do so better than for macaque calls, it would mean that human participants recognize general features in ape vocalizations, but not monkey calls. This would suggest that phylogenetically close species can recognize each other’s calls, even despite their acoustic distance, i.e. acoustic distance can be overcome by evolutionarily-shared perceptual features. In contrast, if only some calls are recognized across species, this would suggest that acoustic features common to all recognized calls would drive recognition. As such, if this were the case, humans should be able to recognize calls according to acoustic features only, and besides primates, open the way for further studies with a larger range of species sharing the highlighted acoustic features.

Overall, it thus remains unclear whether the human ability to recognize affective vocal cues from other species is mainly due to (1) cross-taxa similarities in acoustic parameters, which extends beyond phylogenetic relatedness (2) the phylogenetic closeness between species, which would overcome acoustic distance between the calls or (3) a mix of both. To address these outstanding issues, we designed a forced-choice paradigm, where participants had to perform two tasks: categorization (A versus B, cognitively demanding) and discrimination (A versus non-A; less cognitively demanding). In both tasks, participants were asked to judge the affective nature of vocalizations produced in three affective contexts (threat, distress and affiliation) by humans and three other primate species that vary in phylogenetic proximity to humans (equally close to humans: chimpanzee, bonobo; more distant: rhesus macaque). For each of the two tasks, we measured whether participants were significantly above chance, and whether accuracy of performance could be predicted by stimulus species, affect or their interaction.

To disentangle whether recognition performance was better explained by phylogenetic proximity or acoustic distance, we conducted an acoustic analysis to establish the acoustic distance of chimpanzee, bonobo and macaque vocalizations to human vocalizations. To do so we calculated ‘Mahalanobis distances’ between vocalizations from various affective contexts produced by these species. A Mahalanobis distance is obtained from a generalized pattern analysis computing the distance of each vocalization from the centroids of the different species vocalizations^41^. This analysis allowed us to obtain an acoustic difference matrix used to test how these acoustic differences were differentially related to the human emotional voices. We predicted that if *phylogenetic closeness* was the main determinant of performance, recognition of affective cues in human vocalization should be greater than those of chimpanzees and bonobos, which should be equally better than those of rhesus monkey vocalizations (humans > chimpanzees = bonobos > macaques). By contrast, if *acoustic distance* was the main determinant of performance, participants should perform best with the calls of species most acoustically similar to those of humans. Whilst the phylogenetic closeness hypothesis would predict consistency of performance across affective contexts, it is possible that acoustic distance to human vocalizations may vary with affective context. Therefore, we considered accuracy of performance at each level of affective context separately. As noted above, it is also possible that both phylogenetic closeness and acoustic distance may influence human cross species emotional recognition. If so, we may expect amongst equally related species, more accurate performance with the species with most similar acoustic structures to humans. Thus, if chimpanzees are shown to be more acoustically similar to humans than bonobos overall, or for certain affects, we would expect better recognition accuracy for chimpanzees than bonobos: humans > chimpanzees > bonobos > macaques). Finally, based on the previous literature (Dricu et al. 2017; Gruber et al. 2020), we also expected participants to perform more accurately on discrimination rather than categorization tasks.

## Materials and methods

### Participants

Sixty-eight healthy adult volunteers from the Geneva region, Switzerland (29 males; mean age 23.54 years, SD = 5.09, age range 20–37 years) took part in the experiment. A power analysis calculation revealed that a desired sample size of 66 was required to demonstrate statistical significance of a one sample-study with continuous endpoint in adult humans for the recognition of primate calls. We therefore recruited 68 participants in order to account for possible drop-outs (α = 0.05, β = 0.1, desired power = 0.90, m1 = 0.7, m2 = 0.62). All participants reported normal hearing abilities and normal or corrected-to-normal vision. No participant presented a neurological or psychiatric history, or a hearing impairment. All participants gave informed and written consent for their participation in accordance with the ethical and data security guidelines of the University of Geneva, Switzerland. The study was approved by the Ethics Cantonal Commission for Research of the Canton of Geneva, Switzerland (CCER).

### Vocal stimuli

For our stimuli, we compiled a set of ninety-six vocalizations balanced across four primate species (human, chimpanzee, bonobo, rhesus macaque) and three affective contexts (threat, distress and affiliation). For human stimuli, we obtained non-linguistic vocal stimuli denoted as angry, fearful and happy or from two male and two female actors from the Montreal Affective Voices Audio Collection^42^. For the chimpanzee, bonobo and rhesus macaque stimuli, vocal stimuli of corresponding contexts were taken from existing author databases. For threat, we used aggressor barks in agonistic contexts, for distress, we used victim screams from social conflicts and for affiliation, we used food-grunts. For each species, we selected 24 stimuli, taken from 6 to 8 different individuals; each contained either single calls or two call sequences of a single individual. In line with the previous work^2^, all vocal stimuli were standardized to 750 ms (ms) using PRAAT (www.praat.org) by simply cropping the sounds. Although the stimuli were not normalized for energy to preserve the naturality of the sounds^43^ , the volume of the headphones was fixed at 60% for all participants to minimize amplitude variation.

### Experimental procedure

**Figure 1:**
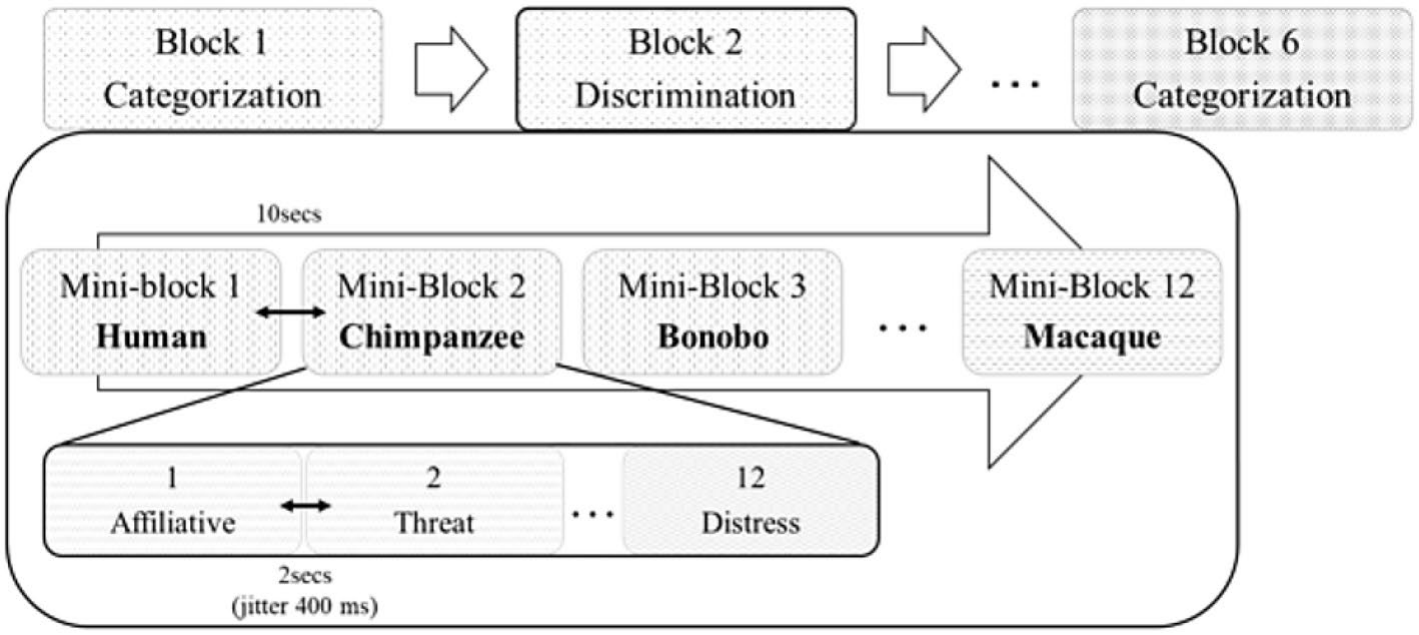
Structure of the experiment, with each of the six blocks made of 12 mini-blocks, which in turn comprised 12 individual trials

Participants were seated in front of a computer while listening to the vocalizations played binaurally using Sennheiser headphones at 70 dB SPL. Each of the 96 stimuli was repeated nine times across six separate counterbalanced blocks, leading to 864 trials following a randomization process. The overall experiment followed a within-subjects design with various layers (Fig. 1). Testing blocks were task-specific: participants either performed a categorization task (A versus B) or a discrimination task (A versus non-A). Participants completed three categorization blocks and three discrimination blocks, resulting in a total of six blocks. Each block was comprised of 144 trials, i.e. 12 mini-blocks, consisting of 12 trials each containing four vocalizations from all three contexts (affiliative/happy; threat/anger; distress/fear) produced by a single species (human, chimpanzee, bonobo, rhesus macaque). The blocks, mini-blocks and stimuli were pseudo-randomly assigned for each participant to avoid more than two consecutive blocks, mini-blocks and stimuli from the same category.

At the beginning of each block, participants were instructed to identify the affective content of the vocalizations using a keyboard. For instance, the instructions for the categorization task could be “Affiliative—press M or Threatening—press Z or Distress—press space bar”. Similarly, the instructions for discrimination could be “Affiliative—press Z or other affect—press M”. The pressed keys were randomly assigned across blocks and participants. The participants pressed the key during 2-s intervals (jittering of 400 ms) between each stimulus. If the participant did not respond during this interval, the next stimulus followed automatically.

### Statistical analysis

#### Acoustic analyses

To quantify the impact of acoustic distance in human affect recognition of primate vocalizations, we automatically extracted 88 acoustic parameters from all stimuli vocalizations using the extended Geneva Acoustic parameters set, which is defined as the optimal acoustic indicators related to human voice analysis (GeMAPS)^44^. This set of acoustical parameters was selected based on (i) their potential to index affective physiological changes in voice production, (ii) their proven value in former studies as well as their automatic extractability, and (iii) their theoretical significance. This set of acoustic parameters includes related frequency parameters (e.g. pitch, jitter, formants), energy parameters (e.g. loudness, shimmer), and spectral parameters (e.g. alpha ratio, Hammarberg index, spectral slopes).

To assess the stimuli acoustic distance, we ran a Discriminant Analysis (DA) using SPSS 26.0.0.0. This DA was based upon the 88 acoustic parameters in order to discriminate our stimuli based on the four different species (human, chimpanzee, bonobo, and rhesus macaque). After excluding the acoustic variables with the highest correlations (> 0.90), we retained 16 acoustic parameters related to frequency, energy, and spectral parameters that could discriminate species (see Supplementary material Table S1).

Using these 16 acoustic features, we subsequently computed Mahalanobis distances of the 96 experimental stimuli. We performed Generalized Linear Mixed Models (GLMMs) to test whether the two fixed factors—species and affect—could predict the Mahalanobis distances from the human centroid. We also examined the interaction between these two factors. The identity of the vocalizer was included as a random factor. The models were fitted by Restricted Maximum Likelihood (REML)—a Likelihood Ratio Test (LRT) statistic—on R.studio^45^ using the package Lme4^46^.

To test the effects of phylogenetic proximity, we performed contrasts of interest on the factor of Species (i.e. human < chimpanzee = bonobo < macaque), taking into account the other fixed and random factors. In order to identify the acoustic similarity between species, we performed relevant pairwise comparisons on Mahalanobis distances from the centroid of human vocalizations: for each affect, we compared: human vs chimpanzee, human vs bonobo; human vs macaque; chimpanzee vs bonobo; chimpanzee vs macaque and bonobo vs macaque. Hence, each subset of data (e.g. threat chimpanzee) appeared a maximum total of 3 times in the pairwise comparisons, leading us to compare our p-values to Bonferroni corrected alpha level of P_corrected_ = 0.05/3 = 0.017.

#### Vocal recognition performance

First, we investigated if recognition accuracy in the categorization and discrimination tasks was significantly above chance for each affect per species (i.e. three affects × 4 species = 12 separate tests for each process). Per participant, we calculated the proportion of correct answers for each affect-species set of calls (N = 8 calls per set) and then used non-parametric tests, i.e. one sample Wilcoxon tests, to examine whether proportion of correct answers was significantly above chance per task (0.33 for categorization task; 0.5 for discrimination task).

Next, to test our hypotheses of phylogenetic proximity (hypothesis 1); acoustic distance (hypothesis 2) or a combination of both (hypothesis 3), we ran GLMMs for both categorization and discrimination tasks separately to examine whether species and affect predicted participant accuracy, expressed as the number of correct answers for each type of stimulus (species*affect e.g. chimpanzee distress). All GLMMs were fitted by REML on R.studio using the “bobyqa” function (optimization by quadratic approximation with a set maximum of 1,000,000 iterations) with the “logit” link for a standard logistic distribution of errors and binomial distribution. We tested the effects of species (human, chimpanzee, bonobo, rhesus macaque) and affect (affiliative, threat, and distress) on the response variable of accuracy in either the discrimination or categorization task. Participant IDs was entered as a random factor. Before interpreting model estimates, we first used likelihood ratio tests to compare all full models against a null model containing only intercept and random effects.

To examine whether species differences matched the pattern predicted by any of our three hypotheses, we ran the same species contrasts, i.e. human vs chimpanzee, human vs bonobo; chimpanzee vs bonobo; chimpanzee vs macaque and bonobo vs macaque for each affect.

## Results

### Acoustic analyses

**Figure 2:**
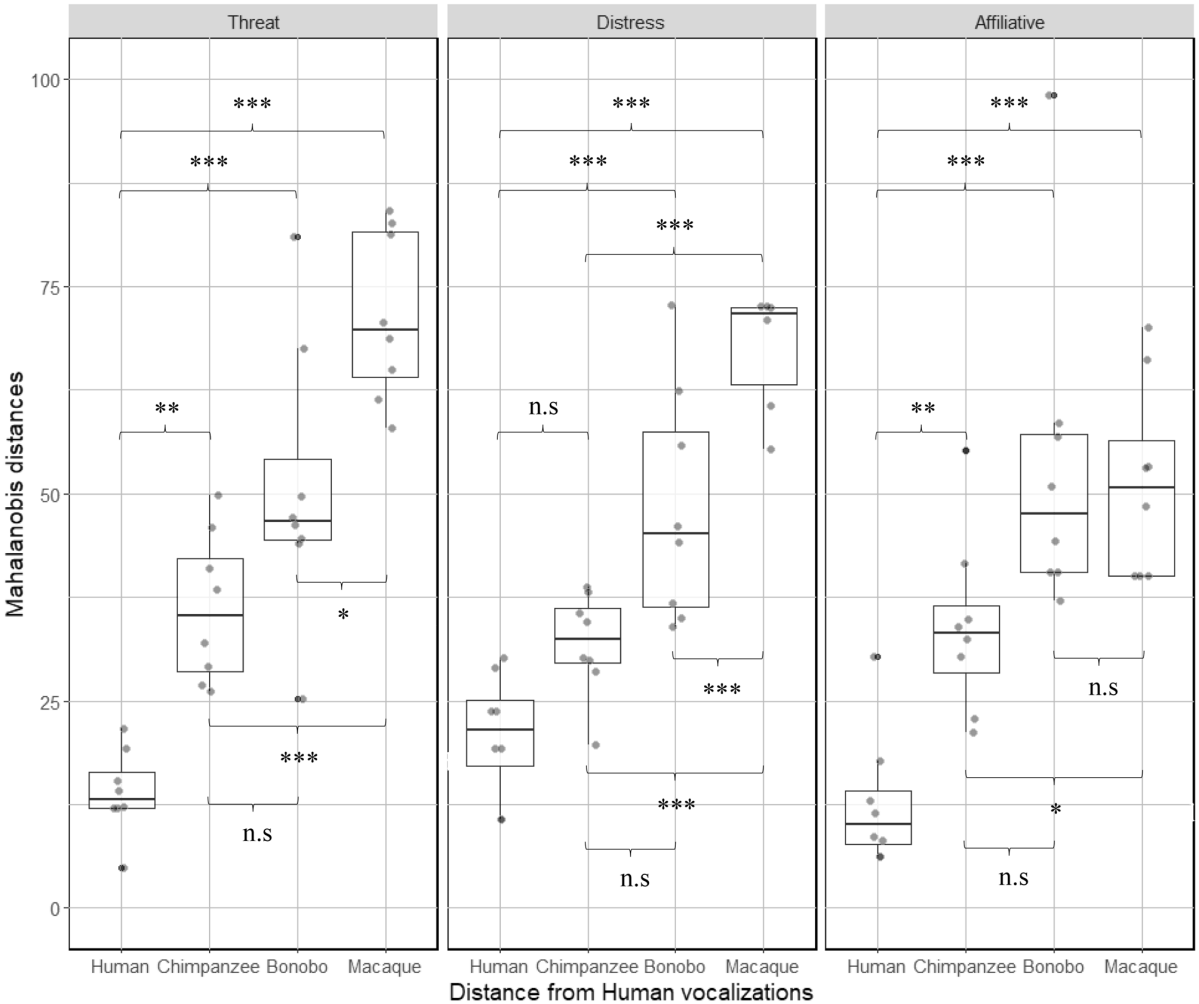
Boxplot of Mahalanobis distances for the 96 vocalizations representing acoustic differences from human voice compared to the other species vocalizations for the different affective states. Higher values represent greater acoustic distances. (* < 0.017; ** < 0.003; *** < *0.0003).* See Table S3 for the detailed results of the post hoc GLMM comparisons.

The DA allowed us to compute Mahalanobis distances for all stimuli as compared to human vocalizations (Fig. 2). A GLMM analysis on Mahalanobis distances revealed that the full model (AIC = 791.88) including main effects explained significantly more variance compared to the null model (AIC = 890.08) with χ^2^(11) = 120.2, *p <* 0.001). The model revealed significant interaction between species and affect (χ^2^(6) = 17.16, *p <* 0.01). Note that affect did not reach significance (χ^2^(2) = 1.68, *p =* 0.431). See Supplementary material Table S2 for model estimates, standards errors and confidence intervals.

To explore the interaction, we examined species differences per level of affect. When corrected for multiple comparisons, pairwise comparisons revealed that Mahalanobis distances to human centroids for human vocalizations were significantly smaller than for all bonobo and all macaque vocalizations, as well as affiliative and threat chimpanzee vocalizations, but not chimpanzee distress calls. Chimpanzee and bonobo vocalizations (when plotted from human vocalization centroids) were not significantly different at the levels of distress and threat. However, bonobo affiliative vocalizations were significantly further from the human centroid than chimpanzee affiliative vocalizations. Macaque vocalizations were significantly further from the human centroid than chimpanzee vocalizations for all three affect categories. Macaque vocalizations were significantly further from the human centroid than bonobo vocalizations for threat and distress calls, but not affiliative calls.

Overall, while the pattern of Mahalanobis distances from the human centroid for threat vocalizations appears to mirror phylogenetic proximity between species (with H > C = B > M), we found significant variation for both distress and affiliative vocalizations. With respect to distress calls, the pattern suggests that great ape calls were acoustically similar to each other, but different from macaque calls (H = C = B > M). In contrast, human affiliative calls were significantly different from all other calls, with chimpanzee calls being significantly closer to the human centroid than bonobo or macaque calls (H > C > B = M). The pairwise comparisons for acoustic differences to the chimpanzee, bonobo or macaque centroid can be found in the Supplementary Material Table S4.

### Vocal recognition performance

Results revealed different patterns of performance for categorization and discrimination tasks, as well the effects of species and affect on task accuracy. In addition, given the different pattern of species differences in acoustic distance from human vocalizations as a function of affect, it was important to consider the recognition data in relation to the affective context as well.

#### Categorization

**Figure 3:**
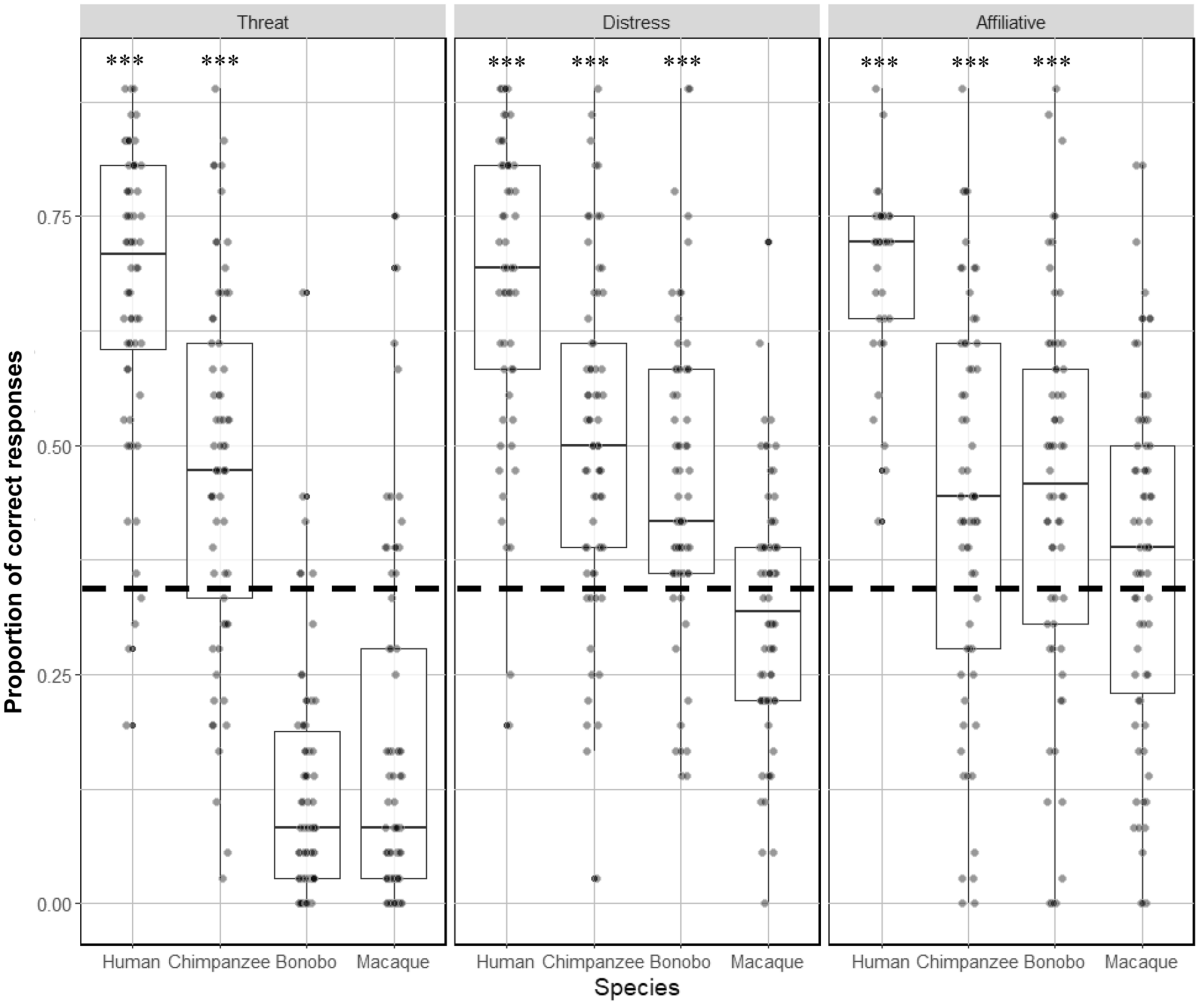
Boxplot illustrating the proportion of correct responses for each category of stimuli in the categorization task. Higher values represent greater accuracy. One sample Wilcoxon analyses against chance level (0.33—represented with the dotted line) are shown. Note that all types of stimuli were categorized at a level significantly above chance, with the exception of all macaque calls and threatening bonobo calls. See Table S5 in Supplementary material for the summary of the one sample Wilcoxon values testing whether participants’ accuracy was above chance level. *** *p <* 0.001.

For all three affective contexts, participant performance was significantly above chance for recognizing the affective context for both human and chimpanzee vocalizations. For bonobo calls, participant performance was only above chance for recognizing distress and affiliative calls, but not threat calls. In contrast, no call type was recognized at significantly above chance levels for macaques (Fig. 3).

For the categorization task, a likelihood ratio test between the full and null model revealed the full model explained a significant amount of variance in the data (χ^2^(11) = 609.3, *p <* 0.001). Within the model, there was a significant interaction (χ^2^ (6) = 17.23, *p <* 0.001) between species and affect.

Contrast analysis revealed that human vocalizations were systematically better recognized than chimpanzee, bonobo and macaque vocalizations across all levels (Table 1). In contrast, accuracy with chimpanzee and bonobos distress and affiliative calls was similar, with chimpanzee threat calls being more accurately categorized than bonobo threat calls. Chimpanzee and bonobo distress and affiliative calls were both more accurately categorized than macaque calls. However, macaque threat calls were more accurately categorized than bonobo threat calls. All contrasts were compared to a corrected P for multiple comparisons (Bonferroni correction: P_corrected_ = 0.05/3 = 0.017).

**Table 1:** Table summarizing the results of post-hoc GLMM pairwise comparisons for categorization across species (chimpanzee, bonobo and macaque) and affect (threat, distress, affiliative. All p-values are compared to a corrected alpha level of 0.017 (* < 0.017; ** < 0.003; *** < *0.0003).* Abbreviations: (Mac) macaque; (Chimp) chimpanzee; (affiliat.) affiliative.

	*Chimp threat*	*Bonobo threat*	*Mac* *threat*		*Chimp distress*	*Bonobo distress*	*Mac* *distress*		*Chimp affiliat*	*Bonobo affiliat*	*Mac affiliat*
*Human threat*	χ^2^(1) = 37.37; *p <* 0.001 ***	χ^2^(1) = 304.97; *p <* 0.001 ***	χ^2^(1) = 252.77; *p <* 0.001 ***	*Human distress*	χ^2^(1) = 44.49; *p <* 0.001 ***	χ^2^(1) = 56.13; *p <* 0.001 ***	χ^2^(1) = 158.69; *p <* 0.001 ***	*Human affiliat*	χ^2^(1) = 132.47; *p <* 0.001 ***	χ^2^(1) = 122.59; *p <* 0.001 ***	χ^2^(1) = 200.93; *p <* 0.001 ***
*Chimp threat*	*–*	χ^2^(1) = 128,84; *p <* 0.001 ***	χ^2^(1) = 95.77; *p <* 0.001 ***	*Chimp distress*		χ^2^(1) = 0.68; *p =* 0.41	χ^2^(1) = 35.13; *p <* 0.001 ***	*Chimp affiliat*	*–*	χ^2^(1) = 0.19; *p =* 0.66	χ^2^(1) = 7.10; *p <* 0.008 *
*Bonobo threat*		*–*	χ^2^(1) = 2.45; *p <* 0.12	*Bonobo distress*		*–*	χ^2^(1) = 26.06; *p <* 0.001 ***	*Bonobo affiliat*		*–*	χ^2^(1) = 9.63; *p <* 0.002 **

#### Discrimination

**Figure 4:**
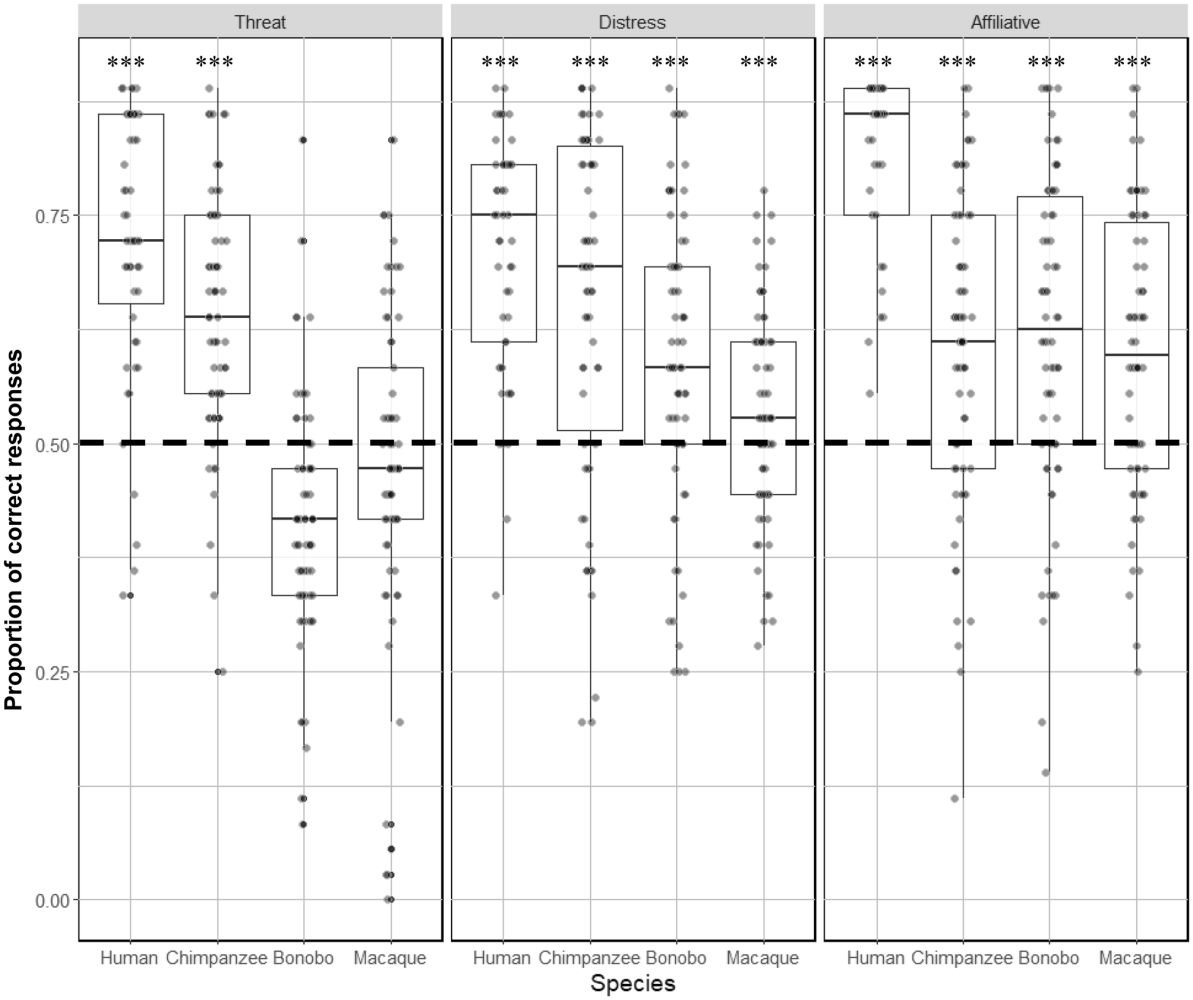
Boxplot illustrating the proportion of correct responses in the discrimination task. Higher values represent greater accuracy. One sample Wilcoxon tests against chance level (0.5—shown with the dotted line) are reported in Table S5 in Supplementary material. Note that all types of stimuli were discriminated at above chance levels with the exception of all macaque calls and threatening bonobo calls. *** *p <* 0.001.

Participants performed significantly above chance when discriminating affect categories for both human and chimpanzee vocalizations. This was also the case for recognizing distress and affiliative calls for bonobos and macaque calls. However, threat calls for the two latter species were not discriminated at a level significantly above chance (Fig. 4).

For the discrimination task, the full model explained the significantly more variation in the data than the null model (χ^2^(11) = 436.97, *p <* 0.001). Within the model, there was a significant interaction between affect and species (χ^2^(6) = 12.23, *p <* 0.001).

Contrast analysis revealed that human vocalizations were systematically better recognized than chimpanzee, bonobo and macaque vocalizations at all levels of affect (Table 2). Chimpanzee threat calls were significantly better discriminated compared to threat calls of both bonobo and macaques, whilst macaque threat calls were better discriminated than bonobo calls. In contrast, while participants were again significantly better at discriminating chimpanzee distress vocalizations than bonobo and macaque distress vocalizations, bonobo distress calls were discriminated better than macaque vocalizations. Finally, none of the contrasts reached significance level for comparison of affiliative vocalizations in non-human primates.

**Table 2:** Table summarizing the results of post-hoc GLMM pairwise comparisons for discrimination across species (chimpanzee, bonobo and macaque) and affect (threat, distress, affiliative. All p-values are compared to a corrected alpha level of 0.017 (* < 0.017; ** < 0.003; *** < *0.0003).* Abbreviations: (Mac) macaque; (Chimp) chimpanzee; (affiliat.) affiliative.

	*Chimp threat*	*Bonobo threat*	*Mac* *threat*		*Chimp distress*	*Bonobo distress*	*Mac* *distress*		*Chimp affiliat*	*Bonobo affiliat*	*Mac affiliat*
*Human* *threat*	χ^2^(1) = 22.96; *p <* 0.001 ***	χ^2^(1) = 202.39; *p <* 0.001 ***	χ^2^(1) = 134.71; *p <* 0.001 ***	*Human distress*	χ^2^(1) = 15.57; *p <* 0.001 ***	χ^2^(1) = 45.77; *p <* 0.001 ***	χ^2^(1) = 83.47; *p <* 0.001 ***	*Human affiliat*	χ^2^(1) = 120.85; *p <* 0.001 ***	χ^2^(1) = 112.96; *p <* 0.001 ***	χ^2^(1) = 128.25; *p <* 0.001 ***
*Chimp threat*	*–*	χ^2^(1) = 89.01; *p <* 0.001 ***	χ^2^(1) = 46.44; *p <* 0.001 ***	*Chimp distress*		χ^2^(1) = 7.95; *p =* 0.004 *	χ^2^(1) = 26.93; *p <* 0.001 ***	*Chimp affiliat*	*–*	χ^2^(1) = 0.13; *p =* 0.72	χ^2^(1) = 0.11; *p =* 0.74
*Bonobo threat*		*–*	χ^2^(1) = 6.86 *p =* 0.009 *	*Bonobo distress*		*–*	χ^2^(1) = 5.62; *p =* 0.018	*Bonobo affiliat*		*–*	χ^2^(1) = 0.49; *p =* 0.49

## Discussion

In this study, we used a combination of acoustic analyses and experimental recognition tasks to investigate how humans perceive primate vocal communication of affect. We compared recognition performance of the vocalizations of two phylogenetically close primates, chimpanzees and bonobos, along with one more distant species; rhesus macaques. The acoustic analysis revealed that affective context modulated the acoustic distance between the human centroid and the calls of chimpanzees, bonobos and rhesus macaques. It was thus critical to consider recognition performance for each affective context individually, rather than across all contexts.

The main aim of the study was to examine if human recognition of affect in other species was better explained by three different hypotheses: *phylogenetic closeness* to the species, *acoustic distance* between the vocalizations of humans and the species in question, or a combination of the two. These three hypotheses generated different patterns of expected effects according to species and affective context (Table 3). We then applied these predictions to the results of human participants in two recognition tasks—discrimination and categorization, measured in two different ways (recognition above chance and performance accuracy). Results showed that none of the human performance patterns extracted from the discrimination task aligned with any of the patterns predicted by the three hypotheses, indicating other factors must be influence performance on this task (Table 3). However, in the categorization task, species differences in accuracy followed the pattern expected by the *phylogenetic closeness* hypothesis for vocalizations produced in both affiliative and distress contexts (Table 3). In contrast, the finding that humans were significantly above chance at categorizing vocalizations in an affiliative context matched the *acoustic distance* hypothesis (Table 3). This mixed pattern of results indicates that human affective recognition performance is influenced by the task type (categorization / discrimination), the measure of performance and the stimuli affective context. Further research needs to consider these factors when trying to further disentangle the relative influence of acoustic distance and phylogenetic closeness on human recognition of cross-species affective content.

**Table 3:** Table summarizing the predictions of the phylogenetic, acoustic and combined predictions and contrasting them with the results in the recognition tasks (H: human; C: chimpanzee; B: bonobo; M: Macaque).

	Threat	Distress	Affiliative
H1 Phylogenetic prediction	H > C = B > M	H > C = B > M	H > C = B > M
H2 Acoustic prediction *(based on Mahalanobis results)*	H > C = B > M	H = C = B > M	H > C > B = M
H3 Combined prediction	H > C = B > M	n/a	n/a
Recognition data – Correspondence with predictions listed above *(PHYLO* = *phylogenetic)*			
Categorization above chance	H = C > B = M	H = C = B > M ACOUSTIC	H = C = B = M
Categorization GLMM	H > C > B = M	H > C = B > M PHYLO	H > C = B > M PHYLO
Discrimination above chance	H = C > B = M	H = C = B = M	H = C = B = M
Discrimination GLMM	H > C > M > B	H > C > B = M	H > C = B = M

Although patterns of results did not match many of the predictions for species differences, there were indications that both the phylogenetic closeness and acoustic distance may have influenced recognition. First, performance with macaque vocalizations was generally poorer than with great ape vocalizations, with participants failing to categorize macaque calls from any context at a level significantly above chance. By contrast, they succeeded with all chimpanzee calls and bonobo calls from two out of three affective contexts. For instance, distress vocalizations and affiliative calls expressed by macaques were often confused by the participants independently of the recognition task (see Figs. S2 and S4 in Supplementary material). Although macaques did not always have greatest acoustic distance from human vocalizations, they are the most distantly related species to humans, indicating phylogenetic proximity may be influential. Second, despite their equal phylogenetic proximity to humans, participants performed better on both tasks with chimpanzee vocalizations than bonobo vocalizations. Similarly to macaques, participants often confused bonobo threat calls with affiliative vocalizations and vice versa (see Fig. S3 in Supplementary material). Bonobo vocalizations were further from the human centroid than chimpanzee vocalizations, although only significantly so in one affective context, raising the possibility that acoustic distance may have influenced the superior performance of participants with chimpanzee compared to bonobo calls.

In terms of the acoustic analyses, the acoustic factors extracted in our Discriminant Analyses revealed that certain acoustic features, such as spectral, frequency, and loudness parameters (see Table S1 in Supplementary material) are important for distinguishing affective vocalizations across different primate species. Perhaps surprisingly, chimpanzees were only significantly closer in acoustic distance to humans as compared to bonobos in the affiliative context, despite most bonobo calls being noticeably higher in pitch than chimpanzee calls. Previous work has shown that bonobo vocalizations are up to an octave higher than chimpanzees, something likely explained by having a shorter larynx^33^. Anecdotally, participants most frequently commented on bonobo vocalizations sounding unusual. It is possible therefore that our objective measure of acoustic distance may not match a more subjective human perception of vocal similarity. We do not know which of the acoustic factors measured are the most attended to by humans; the human perceptual system may weigh the measured parameters differently to the statistical techniques we used. Future work could directly compare objective acoustic measures of acoustic distance with human ratings of perceived similarity or difference, and see if subjective ratings of acoustic similarity predict recognition performance. Further work may also benefit from a more fine-tuned acoustic toolbox adapted to capture salient differences in the vocalizations of human and non-human primate species.

Overall, our study showed that human participants were skilled at recognizing the affective context of non-human primate calls. In the discrimination task, participants performed significantly above chance with some vocalizations of all three species we included. This supports previous research conducted on chimpanzee vocalizations^27, 28^. We have shown for the first time that humans are also capable of discriminating and categorizing bonobo calls produced in some affective contexts. Our results also complement previous research showing highly mixed performance for recognizing the affective nature of rhesus monkey calls^25, 27, 30–32^. Interestingly, our study also underlines the importance of the task in determining performance and indicates the previous mixed findings may be artefacts of task differences. Within our study, participants were significantly above chance in discriminating macaque calls from two out of three contexts, yet not for any of the categorization tasks. This may be due to the existing differences between the two tasks. While in discrimination, participants have to recognize that the calls represent different emotions, in categorization, they have to specifically label the emotional content of the vocalizations. This leads to the fact that categorization is itself more complicated cognitively than discrimination, a phenomenon already described when solely using human emotional calls^7, 8^. Nevertheless, the use of a discrimination forced-choice task yielded some successful recognition of macaque calls, in line with the previous findings of Linnankoski and colleagues^30^, who also used a forced choice paradigm. Studies which failed to find any successful recognition of macaque calls asked participants to rate valence on Likert scales, which may be a more challenging task, where in the face of uncertainty, participants can opt for central options, which fail to discriminate valence accurately.

## Conclusion

Overall, we demonstrated the ability of humans to both categorize and discriminate affective cues in vocalizations of three species of non-human primates. Acoustic analysis revealed that the acoustic distance from chimpanzee, bonobo and macaque vocalizations to human vocalizations varied with affective context. Human affective recognition performance was clearly influenced by the species producing the vocalizations, the affective context in which the vocalization was produced and the task (discrimination / categorization). Although there were some indications that both phylogenetic proximity and acoustic distance were associated with better recognition, no clear support for either of these factors driving recognition performance was obtained. Our study demonstrates that the perception of emotional cues by humans in primate vocalizations is a complex process that does not solely rely on phylogenetic or acoustic distance. Future work should further disentangle the effect of familiarity from potential acoustic parameters. Intriguingly, our findings also suggest that humans might use acoustic distance as one of the ways to infer that a different species is phylogenetically close. Such study would allow, for example, the use of the same and other primates’ calls to address questions related to valence or arousal, but also the use of digitally altered or digitally created calls that could match particular acoustically relevant features, allowing to fine-tune the precise characteristic used by humans to accurately sort calls, at least in the absence of additional perceptual information (e.g. how the animal that produced the vocalization looks like). It would also be interesting to explore neural correlates associated with these phylogenetic and acoustic parameters, to offer another level analysis to the behavioral differences outlined in the present study. Finally, we hope that these new findings will contribute to a better understanding of emotional processing origin in humans, by highlighting where the treatment of both primate and human emotions is similar, and where our own species has differed during its evolution.

## Supplementary Information

Supplementary Information.

## Data Availability

The datasets analyzed during the current study are available in the Yareta repository, https://doi.org/10.26037/yareta:3h7qyho5p5dmtmwgqstii3apgy.

## References

[1] Filippi P (2016). Emotional and interactional prosody across animal communication systems: A comparative approach to the emergence of language. Front. Psychol..

[2] Grandjean D (2005). The voices of wrath: Brain responses to angry prosody in meaningless speech. Nat. Neurosci..

[3] Sander D (2005). Emotion and attention interactions in social cognition: Brain regions involved in processing anger prosody. Neuroimage.

[4] Schore JR, Schore AN (2008). Modern attachment theory: The central role of affect regulation in development and treatment. Clin. Soc. Work J..

[5] Grandjean, D., Bänziger, T. & Scherer, K. R. Intonation as an interface between language and affect. In *Progress in Brain Research* vol. 156, 235–247 (Elsevier, 2006). 10.1016/S0079-6123(06)56012-1 17015083

[6] Scherer K (2003). Vocal communication of emotion: A review of research paradigms. Speech Commun..

[7] Dricu M, Ceravolo L, Grandjean D, Frühholz S (2017). Biased and unbiased perceptual decision-making on vocal emotions. Sci. Rep..

[8] Gruber T (2020). Human Discrimination and categorization of emotions in voices: A functional near-infrared spectroscopy (fNIRS) study. Front. Neurosci..

[9] Brunswick E (1956). Perception and the Representative Design of Psychological Experiments.

[10] Frijda NH (1987). Emotion, cognitive structure, and action tendency. Cogn. Emot..

[11] Frijda NH (2016). The evolutionary emergence of what we call “emotions”. Cogn. Emot..

[12] Gross JJ (1998). The emerging field of emotion regulation: An integrative review. Rev. Gen. Psychol..

[13] Nesse RM (1990). Evolutionary explanations of emotions. Hum. Nat..

[14] Arnal LH, Flinker A, Kleinschmidt A, Giraud A-L, Poeppel D (2015). Human screams occupy a privileged niche in the communication soundscape. Curr. Biol. CB.

[15] Kret ME, Prochazkova E, Sterck EHM, Clay Z (2020). Emotional expressions in human and non-human great apes. Neurosci. Biobehav. Rev..

[16] Darwin, C. The expression of the emotions in man and animals. http://darwin-online.org.uk/content/frameset?pageseq=1&itemID=F1142&viewtype=text (1872).

[17] Morton ES (1977). On the occurrence and significance of motivation-structural rules in some bird and mammal sounds. Am. Nat..

[18] Morton, E. S. Grading, discreteness, redundancy, and motivation-structural rules. In *Acoustic Communication in Birds* 182–212 (Academic Press, 1982).

[19] Anderson DJ, Adolphs R (2014). A framework for studying emotions across phylogeny. Cell.

[20] Filippi P (2017). Humans recognize emotional arousal in vocalizations across all classes of terrestrial vertebrates: Evidence for acoustic universals. Proc. R. Soc. B Biol. Sci..

[21] Slocombe KE, Townsend SW, Zuberbühler K (2009). Wild chimpanzees (*Pantroglodytes schweinfurthii*) distinguish between different scream types: evidence from a playback study. Anim. Cogn..

[22] Gouzoules S (1984). Primate mating systems, kin associations, and cooperative behavior: Evidence for kin recognition?. Am. J. Phys. Anthropol..

[23] Manser MB (2001). The acoustic structure of suricates’ alarm calls varies with predator type and the level of response urgency. Proc. R. Soc. B Biol. Sci..

[24] Gruber T, Grandjean DM (2017). A comparative neurological approach to emotional expressions in primate vocalizations. Neurosci. Biobehav. Rev..

[25] Belin P (2008). Human cerebral response to animal affective vocalizations. Proc. Biol. Sci..

[26] Ferry AL, Hespos SJ, Waxman SR (2013). Nonhuman primate vocalizations support categorization in very young human infants. Proc. Natl. Acad. Sci..

[27] Fritz T (2018). Human behavioural discrimination of human, chimpanzee and macaque affective vocalisations is reflected by the neural response in the superior temporal sulcus. Neuropsychologia.

[28] Kamiloğlu RG, Slocombe KE, Haun DBM, Sauter DA (2020). Human listeners’ perception of behavioural context and core affect dimensions in chimpanzee vocalizations. Proc. R. Soc. B Biol. Sci..

[29] Kelly T (2017). Adult human perception of distress in the cries of bonobo, chimpanzee, and human infants. Biol. J. Linn. Soc..

[30] Linnankoski I, Laakso M, Aulanko R, Leinonen L (1994). Recognition of emotions in macaque vocalizations by children and adults. Lang. Commun..

[31] Scheumann M, Hasting AS, Kotz SA, Zimmermann E (2014). The voice of emotion across species: How do human listeners recognize animals’ affective states?. PLoS ONE.

[32] Scheumann M, Hasting AS, Zimmermann E, Kotz SA (2017). Human novelty response to emotional animal vocalizations: Effects of phylogeny and familiarity. Front. Behav. Neurosci..

[33] Grawunder S (2018). Higher fundamental frequency in bonobos is explained by larynx morphology. Curr. Biol. CB.

[34] Maigrot A-L, Hillmann E, Briefer EF (2022). Cross-species discrimination of vocal expression of emotional valence by Equidae and Suidae. BMC Biol..

[35] Gruber T, Clay Z (2016). A comparison between bonobos and chimpanzees: A review and update. Evol. Anthropol. Issues News Rev..

[36] Greenall JS, Cornu L, Maigrot A-L, de la Torre MP, Briefer EF (2022). Age, empathy, familiarity, domestication and call features enhance human perception of animal emotion expressions. R. Soc. Open Sci..

[37] Briefer, E. Vocal expression of emotions in mammals: Mechanisms of production and evidence. *Commun. Ski.* (2012).

[38] Davila Ross M, Owren MJ, Zimmermann E (2009). Reconstructing the evolution of laughter in great apes and humans. Curr. Biol. CB.

[39] Prüfer K (2012). The bonobo genome compared with the chimpanzee and human genomes. Nature.

[40] Tuttle RH (1993). Kano, T. 1992. The last ape: Pygmy chimpanzee behavior and ecology. Stanford University Press, Stanford, CA, xxviii + 248 pp. ISBN 0-8047-1612-9. Price (hardbound), $45.00. J. Mammal..

[41] Mahalanobis, P. C. On the generalized distance in statistics. In *Proceedings of National Institute of Sciences* vol. 2 49.55 (1936).

[42] Belin P, Fillion-Bilodeau S, Gosselin F (2008). The Montreal Affective Voices: A validated set of nonverbal affect bursts for research on auditory affective processing. Behav. Res. Methods.

[43] Ferdenzi C (2013). Voice attractiveness: Influence of stimulus duration and type. Behav. Res. Methods.

[44] Eyben F (2016). The Geneva minimalistic acoustic parameter set (GeMAPS) for voice research and affective computing. IEEE Trans. Affect. Comput..

[45] Team, R. RStudio: Integrated Development for R. RStudio. (2020).

[46] Bates D, Mächler M, Bolker B, Walker S (2015). Fitting linear mixed-effects models using lme4. J. Stat. Softw..

