# Supplementary material for: Humans recognize affective cues in primate vocalizations: acoustic and phylogenetic perspectives
Source: Sci Rep. 2023 Jul 5;13:10900. doi: 10.1038/s41598-023-37558-3 (PMC10322975; doi:10.1038/s41598-023-37558-3)
Supplement: Supplementary file 1 — Supplementary Information. [file 41598_2023_37558_MOESM1_ESM.docx]

**Supplementary material**

***Discriminant Analysis (DA) and Exploratory Factor Analysis (EFA)***

The DA revealed that 16 acoustical parameters are crucial for discriminating the vocalizations of the different species. An EFA on these parameters showed that most of the variance was explained by Factor 1 (27.14%) and Factor 2 (21.63%) followed by Factor 3 (18.99%), Factor 4 (17.57%) and Factor 5 (16.72%). The highest loadings for Factor 1 is SpectralFlux_sma3_amean (r = 0.723), EquivalentSoundLevel_dBp (r = 0.916) and Loundness_sma3_amean (r = 0.869); for Factor 2 is F2bandwidth_sma3nz_amean (r = 0.789); for Factor 3 is LogReIF0_sma3nz_amean (r = 0.804) and ShimmerLocaldB_sma3nz_amean (r = -0.703); and finally for Factor 5 is SlopeV0-500_sma3nz_amean (r = 0.891) (see Table 1).

*
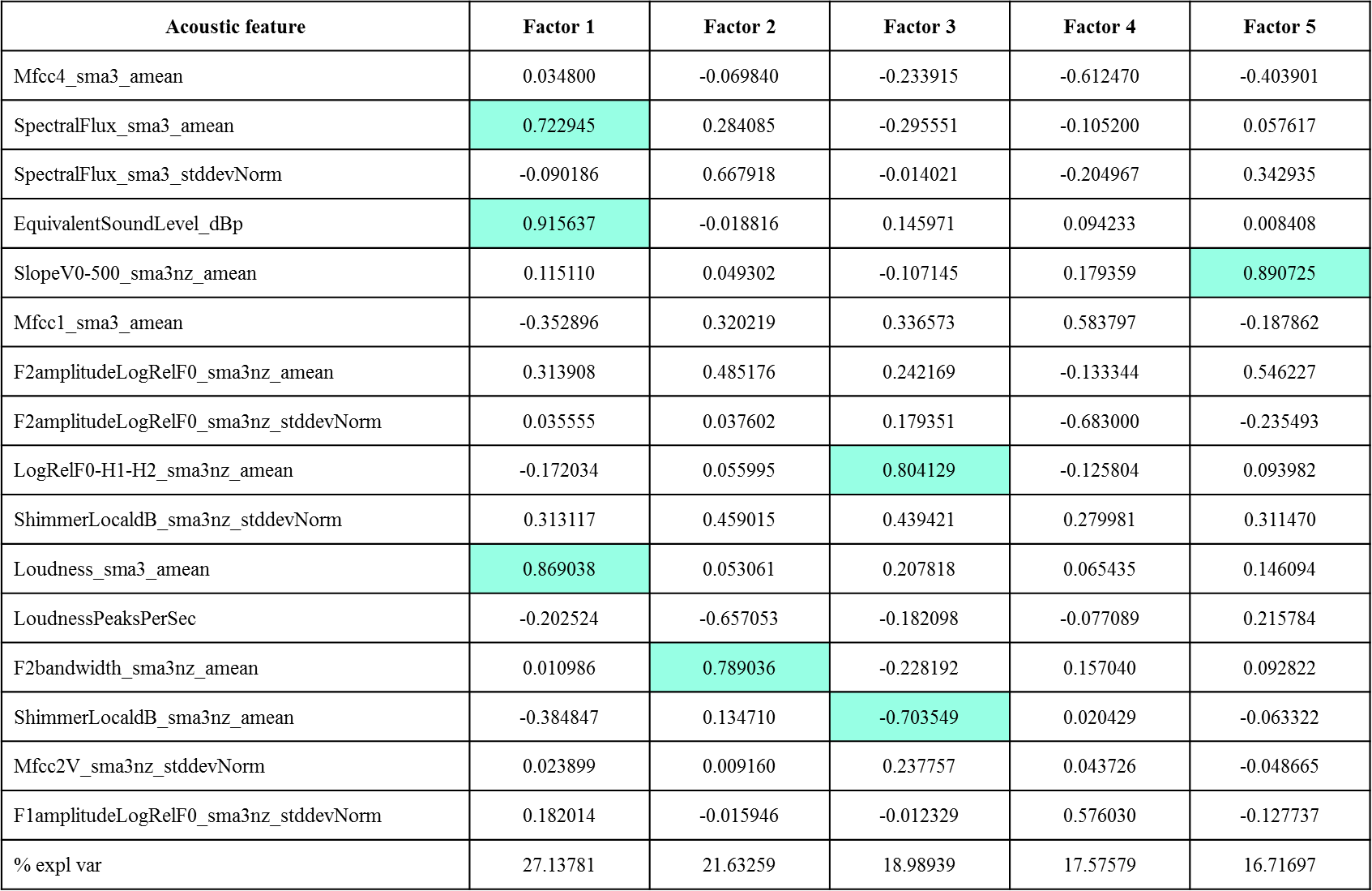
*

Table S1: EFA summary on the acoustical features selected from the GDA and their loadings for the 5 factors discriminating the species across affective contents. Highlighted correlations are > .70. For a full description of the acoustic parameters used see the article Eyben et al., 2016 (Eyben, F., Scherer, K. R., Schuller, B. W., Sundberg, J., André, E., Busso, C., Devillers, L. Y., Epps, J., Laukka, P., Narayanan, S. S., and Truong, K. P. (2016). “The Geneva minimalistic acoustic parameter set (GeMAPS) for voice research and affective computing,” IEEE Trans. Affect. Comput. 7(2), 190–202. <https://doi.org/10.1109/TAFFC.2015.2457417>).

***Mahalanobis distances***

Table S2: Estimates, standard errors (SE) and confidence intervals (lower CI: 2.5% and upper CI: 97.5%) for the GLMM model Species*Affect for acoustic Mahalanobis distances.

| **Term** | **Estimate** | **SE** | **Lower CI** | **Upper CI** |
| --- | --- | --- | --- | --- |
| *Threat* | | | | |
| From human to bonobo | -0.734 | 6.912 | -13.53 | 12.06 |
| From human to chimpanzee | -15.271 | 6.912 | -28.07 | -2.47 |
| From human to macaque | 20.023 | 6.912 | 7.222 | 32.822 |
| *Distress* | | | | |
| From human to bonobo | -3.037 | 6.912 | -15.836 | 9.763 |
| From human to chimpanzee | -19.491 | 6.912 | -32.291 | -6.691 |
| From human to macaque | 29.560 | 6.912 | 16.76 | 45.349 |
| *Affiliative* | | | | |
| From human to bonobo | 1.912 | 6.912 | -10.888 | 14.711 |
| From human to chimpanzee | -17.373 | 6.912 | -30.173 | -4.573 |
| From human to macaque | -38.699 | 6.912 | -51.499 | -25.899 |


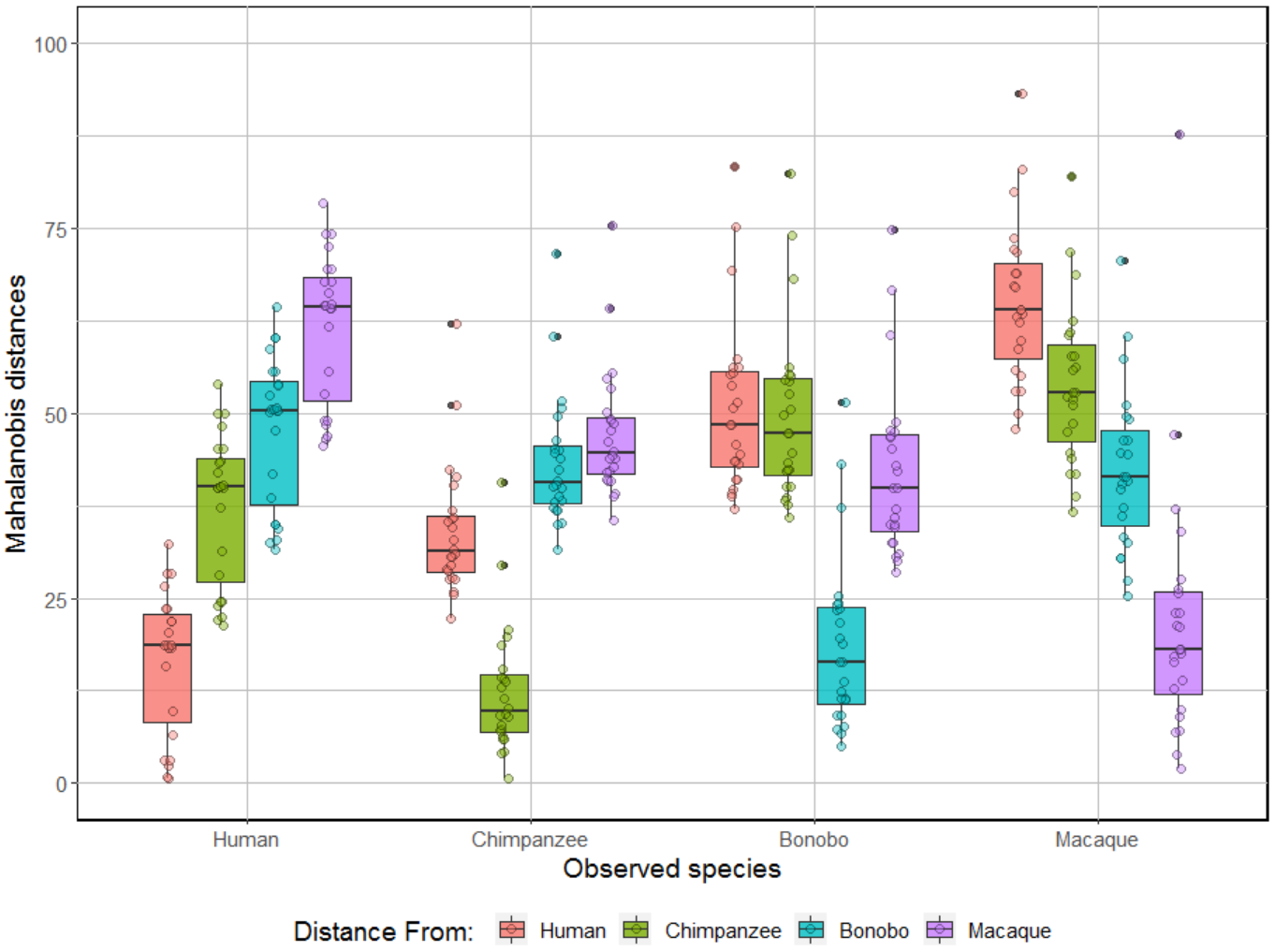


Figure S1: Boxplots of Mahalanobis distances from the GLMM model for the 96 vocalizations representing acoustic distances for each species compared to the others. Higher values represent greater distances. All pairwise comparisons were significant after Bonferroni correction (P_corrected_ = .05/6 = .008), excluding bonobo vs human and bonobo vs macaque from chimpanzee vocalizations; chimpanzee *vs* human/macaque and human *vs* macaque from bonobo vocalizations; and bonobo *vs* chimpanzee from macaque vocalizations (Table S4).

Table S3*:* Table summarizing the results of post-hoc GLMM pairwise comparisons for acoustic differences to the human centroid across species (chimpanzee, bonobo and macaque) and affect (threat, distress, affiliative). All p-values are compared to a corrected alpha level of 0.017 (** <0.017; **<0.003; ***<0.0003).* Abbreviations: (Mac) rhesus macaque; (Chimp) chimpanzee; (affiliat.) affiliative.

|  | ***Chimp threat*** | ***Bonobo threat*** | ***Mac***  ***threat*** |  | ***Chimp distress*** | ***Bonobo distress*** | ***Mac distress*** |  | ***Chimp affiliat.*** | ***Bonobo affiliat.*** | ***Mac***  ***affiliat.*** |
| --- | --- | --- | --- | --- | --- | --- | --- | --- | --- | --- | --- |
| ***Human threat*** | χ^2^(1)=10.3; p=0.001 ** | χ^2^(1)=28.2; p<0.001 *** | χ^2^(1)=69.23; p<0.001 *** | ***Human distress*** | χ^2^(1)=2.58; p=.11 | χ^2^(1)=15.8; p<0.001 *** | χ^2^(1)=75.72; p<0.001 *** | ***Human affiliat.*** | χ^2^(1)=9.52; p=0.002 ** | χ^2^(1)=34.5; p<0.001 *** | χ^2^(1)=31.35; p<0.001 *** |
| ***Chimp threat*** | ***--*** | χ^2^(1)=4.42; p=0.036 | χ^2^(1)=26.0; p<0.001 *** | ***Chimp distress*** |  | χ^2^(1)=5.67; p=0.017 | χ^2^(1)=50.36; p<0.001 *** | ***Chimp affiliat.*** | ***--*** | χ^2^(1)=7.79; p=0.005 * | χ^2^(1)=6.32; p=0.012 * |
| ***Bonobo threat*** |  | ***--*** | χ^2^(1)=9.02; p=0.003 * | ***Bonobo distress*** |  | ***--*** | χ^2^(1)=22.2; p<0.001 *** | ***Bonobo affiliat.*** |  | ***--*** | χ^2^(1)=0.08; p=0.78 |

Table S4: Main contrasts for the Mahalanobis distances analysis on all 96 vocalizations with chi-squared tests and p-values for the interaction Observed-Species and Distance-Species factors (human, chimpanzee, bonobo, rhesus macaque). All p-values are corrected for multiple comparisons using Bonferroni correction (P_corrected_ = .05/6 = .008), in italic the non-significant contrasts.

| Contrasts | χ^2^ | p-values | Contrasts | χ^2^ | p-values |
| --- | --- | --- | --- | --- | --- |
| **Distance from Human voices** |  |  | **Distance from Bonobo vocalizations** |  |  |
| Human vs Chimpanzee | 17.01 | p < 0.001 | Bonobo vs Human | 43.17 | p < 0.001 |
| Human vs Bonobo | 62.74 | p < 0.001 | Bonobo vs Macaque | 36.16 | p < 0.001 |
| Human vs Macaque | 139.36 | p < 0.001 | Bonobo vs Chimpanzee | 30.99 | p < 0.001 |
| Bonobo vs Macaque | 15.09 | p < 0.001 | Chimpanzee vs Human | *1.01* | *p = 0.315* |
| Bonobo vs Chimpanzee | 14.41 | p < 0.001 | Chimpanzee vs Macaque | *0.2* | *p = 0.655* |
| Chimpanzee vs Macaque | 58.99 | p < 0.001 | Human vs Macaque | *0.31* | *p = 0.577* |
| **Distance from Chimpanzee vocalizations** |  |  | **Distance from Macaque vocalizations** |  |  |
| Chimpanzee vs Human | 31.94 | p < 0.001 | Macaque vs Human | 81.59 | p < 0.001 |
| Chimpanzee vs Macaque | 100.76 | p < 0.001 | Macaque vs Chimpanzee | 32.93 | p < 0.001 |
| Chimpanzee vs Bonobo | 71.3 | p < 0.001 | Macaque vs Bonobo | 21.08 | p < 0.001 |
| Bonobo vs Human | *7.8* | *p < 0.01* | Bonobo vs Human | 19.73 | p < 0.001 |
| Bonobo vs Macaque | *2.54* | *p = 0.111* | Bonobo vs Chimpanzee | *1.32* | *p = 0.251* |
| Human vs Macaque | 19.24 | p < 0.001 | Chimpanzee vs Human | 10.85 | p < 0.001 |

***Accuracy***

*Testing whether participants’ accuracy was above chance level*

Table S5: Summary of the one sample Wilcoxon testing if participants’ average accuracy was above the chance level for each species (human, chimpanzee, bonobo and macaque) depending on the affective content of the vocalizations (threat, distress and affiliative) and the recognition task (categorization and discrimination). *** p<0.001. Abbreviations: (Hum) human; (Chimp) chimpanzee; (Bonob) bonobo; (Mac) rhesus macaque.

|  | Threat | | Distress | | Affiliative | |
| --- | --- | --- | --- | --- | --- | --- |
|  | Categorization | Discrimination | Categorization | Discrimination | Categorization | Discrimination |
| Hum | V = 2639949 *** | V = 2218524 *** | V = 2699322 *** | V = 2212522 *** | V = 2842520 *** | V = 2560667 *** |
| Chimp | V = 2173397 *** | V = 1867978 *** | V = 2225685 *** | V = 1925602 *** | V = 2077772 *** | V = 1763535 *** |
| Bonob | V = 673844 p = 1 | V = 1187295 p = 1 | V = 2134554 *** | V = 1720317 *** | V = 2120540 *** | V = 1787545 *** |
| Mac | V = 922064 p = 1 | V = 1378174 p = 1 | V = 1591839 *** | V = 1554648 *** | V = 1801314 *** | V = 1727520 *** |

***Confusion***

threat

Figure S2: Histogram representing the behavioral responses (in percentage) of the participants for affiliative vocalizations expressed by humans, chimpanzees (chimp), bonobos (bonob) and macaques (macaq) independently of the task.

threat

Figure S3: Histogram representing the behavioral responses (in percentage) of the participants for threatening vocalizations expressed by humans, chimpanzees (chimp), bonobos (bonob) and macaques (macaq) independently of the task.

threat

Figure S4: Histogram representing the behavioral responses (in percentage) of the participants for distressful vocalizations expressed by humans, chimpanzees (chimp), bonobos (bonob) and macaques (macaq) independently of the task.
